# Perspectives on adaptive functioning and intellectual functioning measures for intellectual disabilities behavioral research

**DOI:** 10.3389/fpsyg.2023.1084576

**Published:** 2023-03-13

**Authors:** Laura J. Mattie, Susan J. Loveall, Marie Moore Channell, Derek B. Rodgers

**Affiliations:** ^1^Department of Speech and Hearing Science, University of Illinois at Urbana-Champaign, Champaign, IL, United States; ^2^Department of Special Education and Communication Disorders, University of Nebraska-Lincoln, Lincoln, NE, United States; ^3^Department of Teaching and Learning, University of Iowa, Iowa City, IA, United States

**Keywords:** down syndrome, adaptive functioning, intellectual functioning, intellectual disability, methodology in research

## Abstract

**Introduction:**

Intellectual disability (ID) is a significant limitation in both intellectual ability and adaptive functioning, but many studies of participants with ID only include a measure of overall intellectual functioning when describing their samples. The purpose of this perspective article was to provide a starting point for future research regarding the utility of including measures of both intellectual and adaptive functioning in research focused on ID. In this article, we discuss the differences and similarities between the constructs of intellectual and adaptive functioning, how they are measured, and the benefits of using both measures to describe participant abilities. Data are presented to demonstrate that intellectual and adaptive functioning measures capture separate but related skills in a sample of individuals with ID (i.e., children with Down syndrome [DS]; the leading genetic cause of ID).

**Methods:**

Thirty children with DS (7–31 months) were administered the Mullen Scales of Early Learning and their mothers were interviewed using the Vineland Adaptive Behavior Scales.

**Results:**

At the group level, Vineland and Mullen composite scores were relatively normally distributed and positively correlated. At the individual level, a concordance correlation coefficient indicated moderate agreement between Vineland and Mullen composite scores.

**Discussion:**

Although many children showed consistency between measures, others did not. Our discussion and findings, though preliminary, highlight that intellectual and adaptive functioning are separate but related skills and that there are benefits to including both measures when describing samples with ID. We discuss considerations for including adaptive functioning measures to enhance future research on individuals with ID.

## 1. Introduction

Intellectual disability (ID) is defined as significant limitations in intellectual ability (i.e., general mental capacity, IQ) *and* adaptive functioning (i.e., the conceptual, social, and practical skills learned by individuals to participate in everyday life) that emerge during the developmental period (American Psychiatric Association, 2013; World Health Organization, 2018; Schalock et al., 2021). However, many behavioral research studies only include a single measure of IQ when describing samples with ID. This is unfortunate because, unlike IQ, which is believed to remain relatively stable across the lifespan (e.g., Deary, 2014; Schneider et al., 2014; Jenni et al., 2015), adaptive functioning skills increase over time (Tassé et al., 2012, 2016) and provide important information about an individual’s abilities. Thus, the purpose of this perspective article is to present preliminary evidence of the utility of including both measures of intellectual and adaptive functioning in research studies of individuals with ID.

Intellectual and adaptive functioning have a long history of being viewed as related but separate constructs that are influenced by both development and environment (see Keith et al., 1987; Tassé et al., 2016; Tassé and Mehling, 2017). As such, positive correlations have consistently been reported between these measures both in individuals with neurotypical development and those with ID (Carpentieri and Morgan, 1996; Oakland and Harrison, 2011; Sparrow et al., 2016). However, adaptive functioning skills tend to be more concrete (e.g., daily living skills) and translatable to daily life, whereas intellectual functioning skills tend to be more abstract (e.g., reasoning and problem-solving) and academic (Keith et al., 1987; Schalock et al., 2021). Below, we provide overviews of both types of measures and their use in characterizing samples with ID (see also Hamburg et al., 2019).

Intellectual functioning is measured *via* IQ tests. IQ tests are normed with the general population; thus, by definition individuals with ID fall two standard deviations below the mean, representing the tail end of the distribution on the normal curve. Therefore, examination of IQ scores yields limited variability among individuals with ID (i.e., from 70/75 to the floor score, usually around 40, but varies by test). Because IQ tests are intended to represent general intelligence, or “deviation from the norm,” their interpretation generally focuses on what an individual with ID is unable to achieve rather than identifying what they can do (Buntinx, 2013; Thompson et al., 2017). Thus, IQ tests often fail to capture skill gains in individuals with ID. However, because IQ tests provide an interpretable standard score that represents level of cognitive function, they remain widely used to describe samples with ID. To more fully conceptualize ID, we also need information about individuals’ adaptive functioning abilities (Schalock et al., 1994).

Measures of adaptive functioning often rely on informant reports (e.g., caregiver, teacher, self) allowing for a robust description of what an individual can do across functional settings (e.g., home, school, work) rather than the single snapshot of skills captured by an IQ test. Additionally, they provide age-based norms and capture an array of skills starting from birth and extending into adulthood, making floor effects unlikely. These measures aim to identify both an individual’s day-to-day abilities and areas where they require support (Tassé and Mehling, 2017).

Together, intellectual and adaptive functioning measures can provide useful information about an individual’s abilities. Therefore, along with others (Keith et al., 1987; Hamburg et al., 2019), we argue that including an adaptive functioning measure, along with an IQ test, offers several advantages for researchers. First, adaptive functioning measures are less likely than IQ tests to show floor effects (Hamburg et al., 2019). Second, adaptive functioning measures can provide important information about an individual’s functioning in everyday contexts that is not captured by IQ tests (Tassé and Mehling, 2017). Third, the use of caregiver/teacher report of adaptive functioning minimizes the demands on the individual with ID. Thus, the inclusion of adaptive functioning measures enables more holistic and sensitive characterizations of the heterogeneity across individuals with ID, especially for those who are unable to complete IQ tests due to attention difficulties or challenging behaviors.

To demonstrate that intellectual and adaptive functioning measures capture separate but related skills, we present a data example focused on young children with Down syndrome (DS; i.e., the leading genetic cause of ID and one of the most widely investigated etiologies of ID; Mai et al., 2019). Research has documented unique patterns of strength and difficulty (e.g., Daunhauer and Fidler, 2011; Fidler, 2015) along with considerable variability across individuals with DS (Channell et al., 2021). However, even in this “well-studied” population, considerations of both intellectual and adaptive functioning, statistically and/or descriptively, are limited in the research base. Thus, we present data to demonstrate the benefit of including measures of intellectual *and* adaptive functioning in behavioral research. Specifically, we aimed to demonstrate the extent to which these skills are separate and related. Our research questions were:

(1) At the group level, how much variability across the sample is captured by each measure’s standard score, and what is the correlation between those scores?
(2) For each individual, how similar are their standard scores on the two measures?

## 2. Materials and methods

### 2.1. Participants

Participants were 30 young children with DS and their mothers (see Table 1 for demographic information). Data from three studies on DS, all conducted by the first author, were combined for this data example. One study focused on general development from 6 to 24 months, and two studies focused on early language development over 1 year (12–24 and 18–30 months, respectively). Data from children’s first timepoint was used, except for one child who enrolled during the COVID-19 pandemic whose last timepoint was used (when measures could be administered in person). Participants were recruited from the Midwest and Southern regions of the United States through local DS parent groups and early intervention service providers. All participants were reported to have normal or corrected hearing and vision and English as their primary language.

**Table 1:** Demographic information.

	Full sample (*n* = 30)					Vineland-2 (*n* = 13)			Vineland-3 (*n* = 17)		
	*M%*	SD	Range	*Skewness*	Kurtosis	*M%*	SD	Range	*M%*	SD	Range
Child age in months	16.33	6.92	7–31	0.69	−0.42	12.54	5.61	7–24	19.24	6.48	11–31
Mullen-ELC	66.57	11.88	49–93	0.28	−0.44	73.46	10.78	53–93	61.29	10.03	49–82
Vineland-ABC	70.60	9.92	51–85	−0.28	−0.89	75.31	8.52	27–85	67.00	9.59	51–83
Number who scored at floor (*n*)											
Mullen											
ELC	3					0			3		
Visual reception	8					1			8		
Fine motor	8					1			8		
Receptive language	7					1			8		
Expressive language	4					0			4		
Vineland											
ABC	0					0			0		
Communication	1					0			1		
Socialization	0					0			0		
Daily living	0					0			0		
Child sex (% male)	73.3					84.6			64.7		
Child Race/Ethnicity											
White	53.3					38.5			64.7		
Black/African American	6.7					7.7			5.9		
More than 1 Race	16.7					7.7			23.5		
Unknown/Chose not to respond	23.3					46.2			5.9		
Family income											
Under $20,000	0.0					0.0			0.0		
$20,001–$50,000	13.3					15.4			11.8		
$50,001–$100,000	40.1					30.8			47.1		
Over $100,000	26.7					23.1			29.4		
Unknown/Chose not to respond	20.0					30.8			11.8		
Maternal level of education											
Some college	20.0					15.4			23.5		
College degree	23.3					23.1			25.3		
Some graduate training	10.0					15.4			5.9		
Professional/Advanced degree	33.3					23.1			41.2		
Unknown/chose not to respond	13.3					23.1			5.9		

Mullen-ELC = Mullen Early Learning Composite. Vineland-ABC = Vineland Adaptive Behavior Composite. Mullen ELC floor = 49. Mullen domain score floor = 20. Vineland ABC and domain score floor = 20.

### 2.2. Measures

#### 2.2.1. Intellectual functioning

Intellectual functioning was measured using the Mullen Scales of Early Learning (Mullen, 1995). This standardized assessment is normed for ages birth to 68 months and assesses cognition across four domains (visual reception, receptive language, expressive language, fine motor skills), which yield an overall score, the Early Learning Composite (ELC). The ELC has a mean standard score of 100, a standard deviation of 15, and a floor of 49. Each domain yields a standard score with a floor of 20. The Mullen has well-established reliability and validity.

#### 2.2.2. Adaptive functioning

Adaptive functioning was measured using the Vineland Adaptive Behavior Scales (2nd edition, Sparrow et al., 2005; 3rd edition, Sparrow et al., 2016), which examines adaptive functioning across three domains (communication, socialization, daily living skills) and yields an overall score, the Adaptive Behavior Composite (ABC). The ABC has a mean standard score of 100, a standard deviation of 15, and a floor of 20. Each domain yields a standard score with a floor of 20. The Vineland-2/3 is normed from birth through 90+ years.

In our sample, mothers of 13 children completed the Vineland-2 Expanded Interview (Sparrow et al., 2005) because the study was conducted before 2016. Mothers of the other 18 children completed the Vineland-3 Comprehensive Interview (Sparrow et al., 2016). The versions have many overlapping items, but the Vineland-3 was updated to reflect changing cultural demands (e.g., increased use of technology). Both versions have well-established reliability and validity, and strong concurrent validity has been established between them, suggesting high continuity between the two (Sparrow et al., 2016; see Table 1 for information by Vineland version).

### 2.3. Procedure

The University of South Carolina and the University of Illinois Institutional Review Boards approved all study procedures. Mothers provided informed consent. Within the larger assessment batteries, children were administered the Mullen, and mothers were interviewed with the Vineland in the family’s home or at a location near them (e.g., library, DS organization) in person by trained examiners (i.e., the first author or a graduate assistant).

### 2.4. Analytic plan

We conducted both group-level (Research Question 1) and individual-level (Research Question 2) analyses. At the group level, we visualized the distribution of the data and identified the number of children who scored at the floor on composite or domain standard scores. Next, we conducted a paired samples *t*-test with Vineland-ABC and Mullen-ELC scores to determine whether there was a significant difference between the two measures. Last, we computed a Pearson’s *r* correlation coefficient between the Vineland-ABC and Mullen-ELC.

At the individual level, we plotted each child’s Vineland-ABC and Mullen-ELC scores together for visual inspection and calculated a concordance correlation coefficient (CCC) with a 95% confidence interval between the composite scores. The CCC is a measure of agreement between two measures and is expressed as the extent to which the observed data differ from perfect concordance (i.e., a line at 45 degrees on a scatterplot; Lawrence and Lin, 1989). The CCC is a more accurate measure of agreement than a correlation; correlations strictly measure linearity rather than agreement. CCC values are more conservative, as they are the product of the Pearson correlation multiplied by a bias correction factor adjusting for the difference between linearity and agreement (Jinyuan et al., 2016). Values range from –1 to 1, with values closer to 1 indicating stronger agreement between measures. We used Cohen’s (1988) guidelines for interpreting the CCC values (< 0.10 = poor agreement, > 0.30 = moderate agreement, and > 0.50 = high agreement). We used the *epiR* package (Stevenson and Sergent, 2021) in RStudio 1.2.5033 (R Core Team, 2019) to calculate the CCC.

## 3. Results

### 3.1. Group level

The Vineland-ABC and Mullen-ELC scores showed a relatively normal distribution for each measure with no statistical outliers (see Table 1 for skewness and kurtosis; see Supplementary materials for Histograms and Box and Whiskers plots). Three children scored at floor on the Mullen-ELC; no children scored at floor on the Vineland-ABC. Fifteen children scored at floor on one or more domains of the Mullen, with at least one child scoring at floor on every domain. Only one child scored at floor on any domain of the Vineland (i.e., Communication).

A paired-samples *t*-test indicated no significant difference between Mullen-ELC and Vineland-ABC scores, *t* (29) = −1.83, *p* = 0.08, *d* = 0.33. This pattern remained when comparing the Mullen-ELC and Vineland-ABC scores by version (Vineland-2: *t* (12) = −0.60, *p* = 0.56, *d* = 0.17; Vineland-3: *t* (16) = −1.86, *p* = 0.09, *d* = 0.45). A moderate, positive correlation was observed between the Vineland-ABC and Mullen-ELC (*r* = 0.40, *p* = 0.03).

### 3.2. Individual level

**Figure 1:**
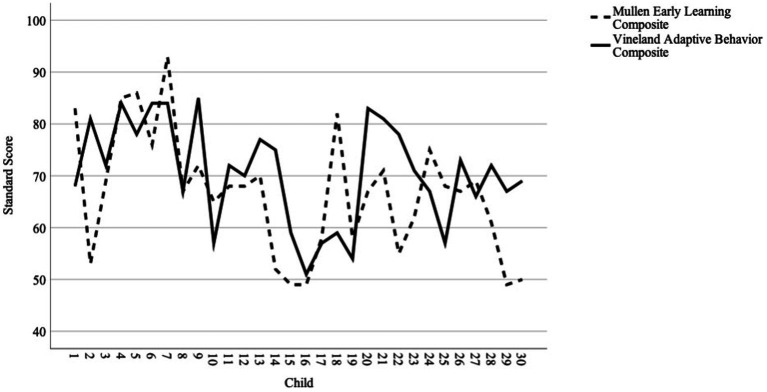
Plot of individual scores on the Vineland-ABC and Mullen-ELC.

Figure 1 shows each child’s score on the Vineland-ABC and Mullen-ELC (see Supplementary Table 1.1 for individual scores and standard errors). Visual inspection indicates consistency between the measures for many children but considerable discord for other children; some scored higher on the Vineland-ABC, and others scored higher on the Mullen-ELC. This was also reflected in the CCC value of 0.38 (CI_95_: 0.05–0.63), indicating moderate agreement (Cohen, 1988). The 95% confidence interval was large, which is likely due, at least in part, to the variability in individuals’ differences between their Vineland-ABC and Mullen-ELC scores.

## 4. Discussion

The purpose of this perspectives article was to present preliminary evidence on the utility of including measures of intellectual and adaptive functioning in research studies. Our data support that intellectual and adaptive functioning are separate but related skills. Although group-level analyses indicated the measures were related (consistent with other studies in DS; e.g., di Nuovo and Buono, 2011; Sabat et al., 2019; Will et al., 2021), analyses at the individual level demonstrated more nuance. Some children scored higher in adaptive functioning, while others scored higher in intellectual functioning. This suggests that intellectual and adaptive functioning measures, or at least the two in our study, capture different skills and involve different demands. These preliminary findings highlight that including both measures when characterizing samples with ID will more fully capture the variability of individual participants and the possible range of abilities across heterogeneous samples (Keith et al., 1987; Karmiloff-Smith et al., 2016).

### 4.1. Considerations for including adaptive functioning measures

Ideally, researchers will include both measures of adaptive and intellectual functioning to more holistically describe their samples with ID because extant data, including the data presented here, indicate they capture different skills (Keith et al., 1987; Hamburg et al., 2019). For example, if the goal is to identify factors contributing significant variance to an outcome, then including a measure of adaptive functioning in addition to intellectual functioning makes sense because they both may contribute unique variance. However, in some situations, researchers may only be able to include one of these measures because of testing length and/or the behavioral demands placed on participants. In this situation, researchers must carefully consider which measure to include because this decision can impact the size and representativeness of the sample depending on who can travel to an assessment site or sit through an assessment session.

There are several situations for which we recommend an adaptive functioning measure instead of an IQ test. For research studies requiring in-person assessments, an adaptive functioning measure may be advantageous when IQ tests are not feasible due to time constraints or participants’ behavioral/attention span limitations. In such cases, if IQ tests are only used as a measure of the level of functioning or control variable, it is difficult to justify an additional 30–60 min for an IQ test when the constructs and outcomes of interest (e.g., language, social skills) must be prioritized. Because it can be completed by a caregiver, the inclusion of an adaptive functioning measure can still provide information on the level of functioning while reducing participant burden.

Although remote-based assessments (e.g., teleassessments) are on the rise and can address travel limitations, IQ tests are just now being translated into this format. Thus, the reliability and validity of IQ teleassessments for populations with ID are unknown, and age-based norms are just being developed. In contrast, adaptive functioning measures are more amenable to online or remote-based studies. These measures are already designed with multiple standardized administration options (in-person, online, or by mailing a hard copy) and do not require the individual with ID to sit through an assessment session. Yet, they still provide valuable information about the individual’s level of functioning, which was demonstrated in our data with Vineland-ABC scores that were normally distributed without floor effects. Thus, we also recommend including a measure of adaptive functioning in remote-based assessment batteries.

One last issue we want to acknowledge is that caregivers may have reservations about IQ tests. Families may have had negative experiences watching their child complete an IQ test or when receiving interpretations of these scores. They may also not want their child’s abilities reduced to a single IQ score. Caregivers therefore may be more likely to consent to their child’s participation in a study that includes an adaptive functioning measure, either in addition to or instead of an IQ test, that allows them to express both their child’s abilities and areas of difficulty.

In sum, the inclusion of an adaptive functioning measure is advantageous to research because it can provide an estimate of level of functioning while circumventing many of the practical limitations associated with research in populations with ID. Even in cases when an IQ test is feasible or preferable, the addition of an adaptive functioning measures can further characterize a sample with ID.

### 4.2. Limitations and future research

Our data are considered preliminary. We used a convenience sample that is not representative of all individuals with DS or ID. We combined data from three pilot studies, as is becoming common practice to achieve larger samples (e.g., shared data repositories). However, due to the timing of these studies, different Vineland versions were administered. Although high continuity has been reported between versions (Sparrow et al., 2016), one study indicated that individuals with intellectual and developmental disabilities may score lower on the Vineland-3 than the Vineland-2 (Farmer et al., 2020). Thus, our findings should be interpreted with caution and warrant replication with the Vineland-3.

Also, the Vineland is based on the caregiver’s interpretation of their child’s behavior rather than elicitation of these behaviors during a structured assessment. Nonetheless, the semi-structured interview approach of the Vineland Expanded/Comprehensive Interview allowed examiners to probe caregiver responses, rather than relying on a checklist, to gather more nuanced information (e.g., if the child can usually do something independently or only sometimes).

Future research should explore the role of intellectual and adaptive functioning measures on developmental outcomes for individuals with ID and when examining phenotypic patterns within (e.g., Fidler et al., 2006) and across (e.g., Burns et al., 2013) neurodevelopmental disorders. For example, Fidler et al. (2006) used the MSEL and VABS to document the early emergence of the “Down syndrome behavioral phenotype,” though they did not compare the two measures. Also, Burns et al. (2013) used the Mullen to assess clinical profiles of cognitive functioning in young children with neurodevelopmental disorders (including autism [*n* = 19], cerebral palsy [*n* = 14], and epilepsy [*n* = 14]). Results showed delays across all domains of the Mullen in children with neurodevelopmental disorders relative to typical development matched on age, gender, and race, but no specific patterns of performance on the Mullen emerged across subgroups. Although Burns and colleagues did not examine the Vineland, it would be informative to see if a similar pattern of results emerged for adaptive functioning in these groups, or if specific patterns may emerge across groups on a functional measure. Larger samples will support the use of more complex analyses, such as latent profile analysis, that can explore these measures in more detail (Channell et al., 2021). Such analyses would also provide insight into individual characteristics that lead to more or less concordance between intellectual and adaptive functioning. Finally, our data focused on young children; future researchers should examine the concordance of adaptive and intellectual functioning measures across the lifespan.

### 4.3. Conclusion

The inclusion of adaptive functioning measures in behavioral research of individuals with ID will help continue the field’s shift from a discussion of deficits toward one focused on strengths and support needs (Burack et al., 2020; Schalock et al., 2021). Based on our preliminary data, we echo Hamburg et al.’s (2019) suggestion that the field would benefit from regularly including measures of adaptive functioning to help describe their samples with ID. This would also allow researchers to descriptively compare intellectual and adaptive functioning skills to see if/how they align or diverge and provide a better understanding of their influence on different outcomes.

## Data availability statement

The raw data supporting the conclusions of this article will be made available by the authors, without undue reservation.

## Ethics statement

The studies involving human participants were reviewed and approved by the University of Illinois at Urbana-Champaign and the University of South Carolina. Written informed consent to participate in this study was provided by the participants’ legal guardian/next of kin.

## Author contributions

LM, SL, and MC conceptualized the study. LM led manuscript drafting. LM and DR conducted the data analysis. LM, SL, MC, and DR edited the manuscript. All authors contributed to the article and approved the submitted version.

## Funding

This research was supported by grants from the ASPIRE grant from the Office of the Vice President for Research at the University of South Carolina (Mattie), the Campus Research Board grant from the Office of the Vice Chancellor for Research at the University of Illinois Urbana-Champaign (Mattie), and the Center on Health, Aging, and Disability’s Pilot Grant Program at the University of Illinois Urbana-Champaign (Mattie).

## Conflict of interest

The authors declare that the research was conducted in the absence of any commercial or financial relationships that could be construed as a potential conflict of interest.

## Publisher’s note

All claims expressed in this article are solely those of the authors and do not necessarily represent those of their affiliated organizations, or those of the publisher, the editors and the reviewers. Any product that may be evaluated in this article, or claim that may be made by its manufacturer, is not guaranteed or endorsed by the publisher.

## References

[ref1] American Psychiatric Association (2013) Diagnostic and statistical manual of mental disorders (DSM-5®). Washington: American Psychiatric Pub.

[ref2] Buntinx W. H. E. (2013). “Understanding disability A strengths-based approach,” in The Oxford Handbook of Positive Psychology. ed. Wehmeyer M. L. (New York, NY: Oxford University Press), 1–22.

[ref3] Burack J. A. Evans D. W. Lai J. Russo N. Landry O. Kovshoff H.. (2020). Edward Zigler’s legacy in the study of persons with intellectual disability: the developmental approach and the advent of a more rigorous and compassionate science. J. Intellect. Disabil. Res. 64, 1–6. doi: 10.1111/jir.12703

[ref4] Burns T. G. King T. Z. Spencer K. S. (2013). Mullen scales of early learning: the utility in assessing children diagnosed with autism spectrum disorders, cerebral palsy, and epilepsy. Appl. Neuropsychol. Child 2, 33–42. doi: 10.1080/21622965.2012.682852 23427775

[ref5] Carpentieri S. Morgan S. B. (1996). Adaptive and intellectual functioning in autistic and nonautistic retarded children. J. Autism Dev. Disord. 26, 611–620. doi: 10.1007/BF02172350, PMID: 8986847

[ref6] Channell M. M. Mattie L. J. Hamilton D. R. Capone G. T. Mahone E. M. Sherman S. L.. (2021). Capturing cognitive and behavioral variability among individuals with down syndrome: a latent profile analysis. J. Neurodev. Disord. 13. doi: 10.1186/s11689-021-09365-2 PMC8056665 33874886

[ref7] Cohen J. (1988) Statistical power analysis for the behavioral sciences. 2nd. Hillsdale, NJ: Erlbaum.

[ref8] Daunhauer L. A. Fidler D. J. (2011). The down syndrome behavioral phenotype: implications for practice and research. Occup. Ther. Health Care 25, 7–25. doi: 10.3109/07380577 23898980

[ref9] Deary I. J. (2014). The stability of intelligence from childhood to old age. Curr. Dir. Psychol. Sci. 23, 239–245. doi: 10.1177/0963721414536905

[ref10] di Nuovo S. Buono S. (2011). Behavioral phenotypes of genetic syndromes with intellectual disability: comparison of adaptive profiles. Psychiatry Res. 189, 440–445. doi: 10.1016/j.psychres.2011.03.015 21507490

[ref11] Farmer C. Adedipe D. Bal V. H. Chlebowski C. Thurm A. (2020). Concordance of the Vineland adaptive behavior scales, second and third editions. J. Intellect. Disabil. Res. 64, 18–26. doi: 10.1111/jir.12691, PMID: 31657503 PMC6941197

[ref12] Fidler D. J. (2015). Early Intervention in Down Syndrome: Targeting the Emerging Behavioral Phenotype. Perspectives on Language Learning and Education 16, 83–89. doi: 10.1044/lle16.3.83

[ref13] Fidler D. J. Hepburn S. L. Rogers S. (2006). Early learning and adaptive behaviour in toddlers with down syndrome: evidence for an emerging behavioural phenotype? Downs Syndr. Res. Pract. 9, 37–44. doi: 10.3104/reports.297 16869373

[ref14] Hamburg S. Lowe B. Startin C. M. Padilla C. Coppus A. Silverman W.. (2019). Assessing general cognitive and adaptive abilities in adults with down syndrome: a systematic review. J. Neurodev. Disord. 11, 1–16. doi: 10.1186/s11689-019-9279-8, PMID: 31470792 PMC6716931

[ref15] Jenni O. G. Fintelmann S. Caflisch J. Latal B. Rousson V. Chaouch A. (2015). Stability of cognitive performance in children with mild intellectual disability. Dev. Med. Child Neurol. 57, 463–469. doi: 10.1111/dmcn.12620, PMID: 25363202

[ref16] Jinyuan L. I. U. Wan T. Guanqin C. Yin L. U. Changyong F. (2016). Correlation and agreement: overview and clarification of competing concepts and measures. Shanghai Arch. Psychiatry 28:115. 27605869 10.11919/j.issn.1002-0829.216045 PMC5004097

[ref17] Karmiloff-Smith A. Al-Janabi T. D’Souza H. Groet J. Massand F. Mok K.. (2016). The importance of understanding individual differences in down syndrome. F1000 Res. 5, 1–10. doi: 10.12688/f1000research.7506.1 PMC4806704 27019699

[ref18] Keith T. Z. Fehrmann P. G. Harrison P. L. Pottebaum S. M. (1987). The relation between adaptive behavior and intelligence: testing alternative explanations. J. Sch. Psychol. 25, 31–43. doi: 10.1016/0022-4405(87)90058-6

[ref19] Lawrence I. Lin K. (1989). A concordance correlation coefficient to evaluate reproducibility. Biometrics 45, 255–268. 2720055

[ref20] Mai C. T. Isenburg J. L. Canfield M. A. Meyer R. E. Correa A. Alverson C. J.. (2019). National Birth Defects Prevention Network (2019) national population-based estimates for major birth defects, 2010–2014. Birth Defect. Res. 111, 1420–1435. doi: 10.1002/bdr2.1589, PMID: 31580536 PMC7203968

[ref21] Mullen E. (1995) Mullen Scales of Early Learning. Circle Pines, MN: American Guidance Service.

[ref22] Oakland T. Harrison P. L. (2011) Adaptive Behavior Assessment System-II: Clinical Use and Interpretation. Washington: Academic Press.

[ref23] R Core Team (2019) ‘R: A Language and Environment for Statistical Computing.’ Vienna, Austria: R foundation for statistical computing.

[ref24] Sabat C. Tassé M. Tenorio M. (2019). Adaptive behavior and intelligence in adolescents with down syndrome: an exploratory investigation. Intellect. Dev. Disabil. 57, 79–94. doi: 10.1352/1934-9556-57.2.79 30920913

[ref25] Schalock R. L. Luckasson R. Tassé M. J. (2021) Intellectual Disability: Definition, Diagnosis, Classification, and Systems of Supports. Silver Spring, MD, US: AAIDD. 10.1352/1944-7558-126.6.439 34700345

[ref26] Schalock R. L.. (1994). The changing conception of mental retardation: implications for the field. Ment. Retard. 32:181. 8084269

[ref27] Schneider W. Niklas F. Schmiedeler S. (2014). Intellectual development from early childhood to early adulthood: the impact of early IQ differences on stability and change over time. Learn. Individ. Differ. 32, 156–162. doi: 10.1016/j.lindif.2014.02.001

[ref28] Sparrow S. S. Cicchetti D. Balla D. A. (2005) Vineland-II: Vineland adaptive behavior scales, 2nd. Minneapolis, MN: Pearson.

[ref29] Sparrow S. S. Cicchetti D. V. Saulnier C. A. (2016) Vineland-3: Vineland adaptive behavior scales. PsychCorp. 10.1093/jpepsy/10.2.215 4020603

[ref30] Stevenson M. Sergent E. (2021) ‘epiR: tools for the analysis of epidemiological data’. Available at: https://cran.r-project.org/package=epiR (Accessed July 8, 2022).

[ref31] Tassé M. J. Luckasson R. Schalock R. L. (2016). The relation between intellectual functioning and adaptive behavior in the diagnosis of intellectual disability. Intellect. Dev. Disabil. 54, 381–390. doi: 10.1352/1934-9556-54.6.381 (Accessed July 8, 2022). 27893317

[ref32] Tassé M. J. Mehling M. H. (2017). “Measuring intellectual functioning and adaptive behavior in determining intellectual disability” in Handbook of Research-Based Practices for Educating Students with Intellectual Disability. (New York, NY: Routledge), 63–78.

[ref33] Tassé M. J. Schalock R. L. Balboni G. Bersani H. Borthwick-Duffy S. A. Spreat S.. (2012). The construct of adaptive behavior: its conceptualization, measurement, and use in the field of intellectual disability. Am. J. Intellect. Dev. Disabil. 117, 291–303. doi: 10.1352/1944-7558-117.4.291, PMID: 22809075

[ref34] Thompson J. R. Shogren K. A. Wehmeyer M. L. (2017). “Supports and support needs in strengths-based models of intellectual disability” in Handbook of research-based practices for educating students with intellectual disability. (New York, NY: Routledge), 17–30.

[ref35] Will E. A. Schworer E. K. Esbensen A. J. (2021). The role of distinct executive functions on adaptive behavior in children and adolescents with down syndrome. Child Neuropsychol. 27, 1–19. doi: 10.1080/09297049.2021.1917531 33938385 PMC8484022

[ref36] World Health Organization (2018) International classification of diseases for mortality and morbidity statistics (11th revision) Geneva: World Health Organization.

