# Supplementary figures and images for: Perspectives on adaptive functioning and intellectual functioning measures for intellectual disabilities behavioral research
Source: Front Psychol. 2023 Mar 13;14:1084576. doi: 10.3389/fpsyg.2023.1084576 (PMC10040585; doi:10.3389/fpsyg.2023.1084576)

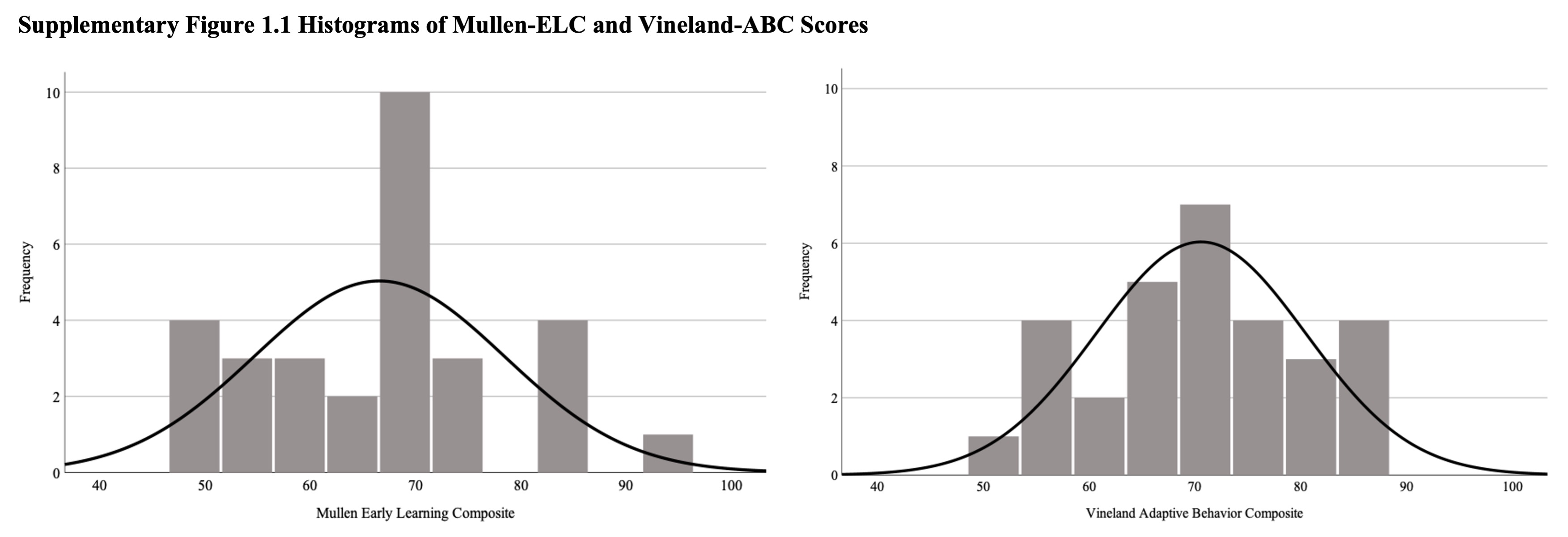

Supplement: Supplementary file 1 [file Image_1.JPEG]

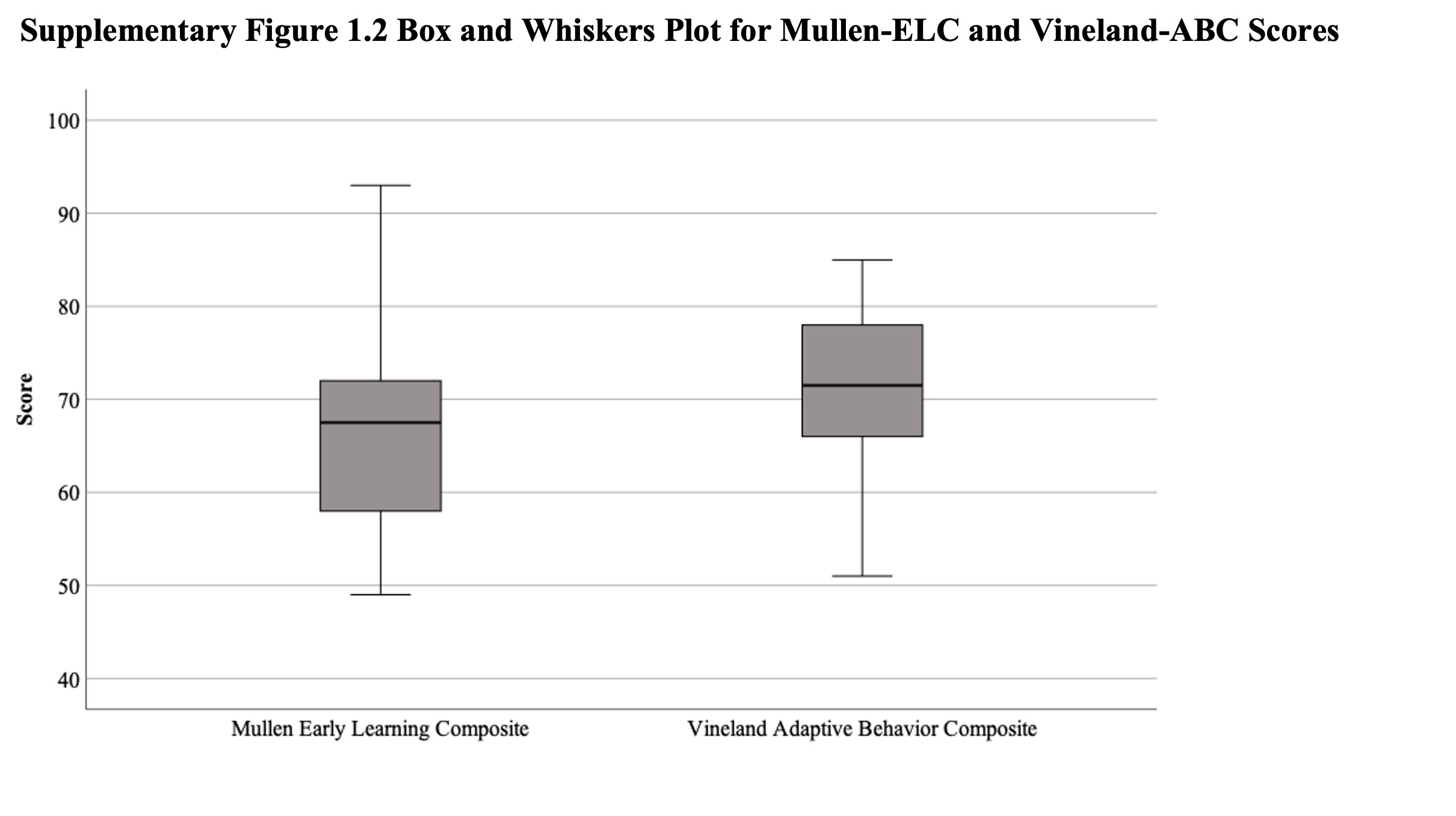

Supplement: Supplementary file 2 [file Image_2.JPEG]
