# Supplementary material for: Perspectives on adaptive functioning and intellectual functioning measures for intellectual disabilities behavioral research
Source: Front Psychol. 2023 Mar 13;14:1084576. doi: 10.3389/fpsyg.2023.1084576 (PMC10040585; doi:10.3389/fpsyg.2023.1084576)
Supplement: Supplementary file 3 [file Table_1.DOCX]

# Supplementary Table 1.1 Individual Participant Mullen-ELC and Vineland-ABC with Standard Error

| *Participant* | *Mullen* | | *Vineland* | |
| --- | --- | --- | --- | --- |
|  | *ELC* | *SE* | *ABC* | *SE* |
| 1* | 83 | 80.83-85.17 | 68 | 66.19-69.81 |
| 2* | 53 | 50.83-55.17 | 81 | 79.19-82.81 |
| 3* | 69 | 66.83-71.17 | 72 | 70.19-73.81 |
| 4* | 85 | 82.83-87.17 | 84 | 82.19-85.81 |
| 5* | 86 | 83.83-88.17 | 78 | 76.19-79.81 |
| 6* | 76 | 73.83-78.17 | 84 | 82.19-85.81 |
| 7* | 93 | 90.83-95.17 | 84 | 82.19-85.81 |
| 8* | 67 | 64.83-69.17 | 67 | 65.19-68.81 |
| 9* | 72 | 69.83-74.17 | 85 | 83.19-86.81 |
| 10* | 65 | 62.83-67.17 | 57 | 55.19-58.81 |
| 11* | 68 | 65.83-70.17 | 72 | 70.19-73.81 |
| 12* | 68 | 65.83-70.17 | 70 | 68.19-71.81 |
| 13* | 70 | 67.83-72.17 | 77 | 75.19-78.81 |
| 14^ | 52 | 49.83-54.17 | 75 | 73.19-76.81 |
| 15^ | 49 | 46.83-51.17 | 59 | 57.19-60.81 |
| 16^ | 49 | 46.83-51.17 | 51 | 49.19-52.81 |
| 17^ | 58 | 55.83-60.17 | 57 | 55.19-58.81 |
| 18^ | 82 | 79.83-84.17 | 59 | 57.19-60.81 |
| 19^ | 58 | 55.83-60.17 | 54 | 52.19-55.81 |
| 20^ | 67 | 64.83-69.17 | 83 | 81.19-84.81 |
| 21^ | 71 | 68.83-73.17 | 81 | 79.19-82.81 |
| 22^ | 55 | 57.17-52.83 | 78 | 76.19-79.81 |
| 23^ | 62 | 59.83-64.17 | 71 | 69.19-72.81 |
| 24^ | 75 | 72.83-77.17 | 67 | 65.19-68.81 |
| 25^ | 68 | 65.83-70.17 | 57 | 55.19-58.81 |
| 26^ | 67 | 64.83-69.17 | 73 | 71.19-74.81 |
| 27^ | 69 | 66.83-71.17 | 66 | 64.19-67.81 |
| 28^ | 61 | 58.83-63.17 | 72 | 70.19-73.81 |
| 29^ | 49 | 46.83-51.17 | 67 | 65.19-68.81 |
| 30^ | 50 | 47.83-52.17 | 69 | 67.19-70.81 |
